# 

**Published:** 1822-12-01

**Authors:** 


### BIBLIOGRAPHICAL RECORD;

OR,

### *Books received for Review within the last Quarter.*

1. Tentamen Medicum inaugurale de Choleræ Morbi, qualis presertim in Orientis Indie erat Causa, Proxima, atque Pathologia Inquisitionem Complectens. By JOHN FAWCETT, M.D. Assistant Surgeon of the 24th Regiment.

☞ *A good deal of ingenious theory is brought into action in this little thesis.*

---

2. Dissertatio Medica Inauguralis quædam de Febre sub Tropicis Regionibus accedente complectens. By PATRIC M'TERNAN, M.D. Surgeon in the Royal Navy.

☞ *Dr. M'Ternan shews the fatal consequences of pursuing the Brunonian System, which happened under his own eyes on the Coast of Africa; and the good effects of the antiphlogistic method of treatment in the West Indies, Bermuda, &c.*

---

3. De la Ligature de l'Artere dans l'Operation de l'Aneurisme par la Methode Moderne; Thèse préséntée et Soutenue à la Faculté de Medecine de Paris, le 25 Juillet, 1822. Par AUG. PECOT, de Besançon, Docteur en Medecine, Ex-chirurgien interne de l'Hôpital civil de Besançon, &c. Quarto, pp. 62, with plates. Paris, 1822. *Presented through M. Breschet, M. D. &c.*

---

4. REVUE MEDICALE, &c. for May, June, and July, 1822.

---

5. Discours prononcé par M. LE BARON DUPUYTREN, President de la Faculté de Medecine de Paris. Seance Publique de la Faculté de Medecine de Paris, du 22 November, 1821. Quarto, Paris, 1822. Also, Discours Prononcé, par M. LE BARON CUVIER.

☞ *Baron Dupuytren's "Discours" contains a long, and not inelegant* ELOGE *on the character and talents of Richard the Naturalist, and Corvisart the Physician, both lately deceased. He also makes some observations on the "Concours" or mode of electing the professors in the Ecole de Medecine, which appears, like many other things, beautiful in theory, but full of difficulties and imperfections when reduced to practice. We shall, in a future number, introduce some biographical notices of Corvisart from Baron Dupuytren's Discourse. Baron Cuvier's Discourse only occupies a couple of pages; but it is full of sentiment, and somewhat tinged with melancholy—especially where he touches on man—"cet etre incomprehensible, melange surprenant des natures les plus contraires, jouet perpetuel des forces les plus opposes."*

6. Inquiry respecting Mr. Charles Whitlaw's Practice in Scrofula and Cancer, and the propriety of instituting an asylum under his care for these complaints, &c. By A. Rennie, Surgeon. Octavo, pp. 38, sewed. Sept. 1822.

☞ *We are surprised that Mr. Rennie should give himself so much trouble in counteracting quackery, for he must be aware that it is out of his power to curtail John Bull's darling privilege of being gulled by every impostor. The enlightened Cockneys who form Mr. Whitlaw's committee and patrons, at present, are soon destined, in their turn, to be objects at which the finger of ridicule will be pointed by their neighbours. The poor wretches now labouring under disease will, it is true, be the greatest sufferers, and they will have ample cause to imprecate retributive justice on the heads of their betters, who are leading them astray—heads, however, which combine the contrasting qualities of being at once as empty of sense as Yorick's in the scene of the grave-diggers—and as impenetrable to knowledge as Memnon's in the British Museum.*

*We are* not *surprised to find the author of the "Philosophy of Medicine" prostituting the few [alas! very few] talents he possesses in aiding any cause good, bad, or indifferent, for he has long been lost to a proper sense of medical decorum; but we* are *surprised that the College of Physicians should permit him thus to lessen the dignity of the medical profession. Grey hairs and even poverty are no excuse for a man's disgracing the corps of which he may be an unworthy member.*

---

7. Elements of Therapeutics and Materia Medica. To which are prefixed two Discourses on the History and Improvement of the Materia Medica, originally delivered as Introductory Lectures. By N. Chapman, M.D. Professor of the Institutes and Practice of Physic, and Clinical Practice in the University of Pensylvania; President of the Academy of Medicine of Philadelphia, &c. Two volumes, 8vo, pp. 420 and 534. Philadelphia, 1821. *Second Edition,* revised and enlarged.

☞ *We hope soon to have the pleasure of presenting the European reader with an analytical account of these valuable productions of the transatlantic press.*

---

8. An Inquiry into the Action of Mercury on the Living Body. By Joseph Swan, Member of the Royal College of Surgeons, and Surgeon to the Lincoln County Hospital. Octavo, sewed, pp. 30. London, 1822.

---

9. Of the Nerves which associate the Muscles of the Chest, in the Actions of Breathing, Speaking, and Expression; being a continuation of the paper on the Structure and Functions of the Nerves. By Charles Bell, Esq. (From the Philosophical Transactions.) Quarto, with a plate, 1822.

---

10. Note sur deux Enfans Nouveau-nés, Hydrocephales et Manquant de Cerveau. Par J. Breschet, D.M. &c.

☞ *In the first case the child lived two days after its deposition in the Hospice des Enfans Trouvées, and appeared to be ten or twelve days old, when left there. The whole of the cerebrum was wanting—its place being filled with serum.*

11. Revue Medicale, for August 1822. Edited by Dr. Amédée Dupau.

☞ *Dr. A. will see that we have attended to the arrangement which he has pointed out to us.*

---

12. Anatomical and Physiological Commentaries. By Herbert Mayo, Surgeon, and Lecturer in Anatomy. No. 1. August, 1822. With eight lithographic plates. Octavo, pp. 120.

☞ *This first number contains—1. Introductory Observations on the Vital Principle; 2. Experiments illustrating the Phenomena of Muscular Action; 3. Reil's Essays on the Structure of the Brain. 4. Experiments to determine the Influence of the Portio Dura of the seventh, and of the Facial Branches of the Fifth Pair of Nerves.*

*Mr. Mayo is a young gentleman of acknowledged talents, and will make a physiological and anatomical journal both instructive and entertaining, if he takes pains. We hope to be able soon to give an account in detail of this first number; but in the mean time we consider that the undertaking deserves the patronage of the public—and especially of medical societies and book-associations.*

---

13. A Review of some of the General Principles of Physiology, with the Practical Inferences to which they have led. By A. P. W. Philip, M.D. F.R.S. E. Octavo, pp. 58. (From the Journal of Science )

---

14. Observations on the Effects of Mercury on the Organs of Hearing, and the improper Use of it in Cases of Nervous Deafness. By W. Wright, Surgeon and Aurist to her late Majesty. Octavo, sewed, pp. 24. Callow and Wilson, 1822.

---

15. Tentamen Medicum Inaugurale de Hygeia. Auctore Ricardo Abell, M.D. Ed. 1822.

---

16. Practical Observations on Distortions of the Spine, Chest, and Limbs; together with Remarks on Paralytic and other Diseases connected with impaired or defective Motion. By William Tilleard Ward, F. L. S. Member of the Royal College of Surgeons, of the Medico-Chirurgical Society, and Fellow of the Medical Society of London. One volume, 8vo, pp. 168. London, 1822.

---

17. A Treatise on Dislocations and Fractures of the Joints. By Sir Astley Cooper, Bart. F. R. S. Surgeon to the King, &c. &c. &c. Quarto, pp. 562, and 30 plates, price *one guinea and a half.* London, October 1822.

---

18. The Philadelphia Journal of the Medical and Physical Sciences, No. 8, for August 1822.

☞ *We are glad to learn from this number of our respected cotemporary that two new medical journals have been recently added to the list of American periodicals—one at Cincinnati, beyond the Alleghany Mountains—the other at New York. We wish them success, and invite their editors to a reciprocal exchange. If the Cincinnati journalists can point out any channel through which we can transmit them our Review, we shall not fail in punctuality. We have transmitted the present number to the editors of the New York Medical and Surgical Journal.*

19. A New View of the Infection of Scarlet Fever, illustrated by Remarks on other Contagious Disorders. By WILLIAM MACMICHAEL, M. D. F. R. S. Fellow of the Royal College of Physicians, &c. and one of the Physicians to the Middlesex Hospital. Octavo, pp. 100. London, October 1822.

---

20. On the Mechanism of the Spine. By HENRY EARLE, Esq. F. R. S. Surgeon to the Foundling, and Assistant Surgeon to St. Bartholomew's Hospital. (From the Philosophical Transactions.) Quarto, pp. 8, with a plate.

☞ *This paper affords additional proofs of the astonishing wisdom and design with which every part of every animal is adapted to the function it is to perform.*

---

21. The Way to preserve good Health, invigorate a delicate Constitution, and attain an advanced Age; together with a Treatise on Domestic Medicine; pointing out, in plain language, the Nature, Symptoms, Causes, probable Terminations, and Treatment of all Diseases incident to Men, Women, and Children, in both Cold and Warm Climates; as also appropriate Prescriptions in English, &c. &c. By ROBERT THOMAS, M. D. &c. Octavo, pp. 707. London, 1822.

---

22. An Introduction to the Study of Fossil Organic Remains, especially of those found in the British Strata: intended to aid the Student in his Enquiries respecting the Nature of Fossils, and their Connexion with the Formation of the Earth. By JAMES PARKINSON, Fellow of the Royal College of Surgeons, Member of the Geological Society of London, &c. One volume, 8vo, pp. 346, with ten copper-plates, containing a great number of figures—price 12s. in boards. Sherwood and Co. 1822.

☞ *This little work contains a great mass of curious research and most interesting details. It is an attempt to shew the difference of forms and structure in the numerous organized beings with which the earth was peopled before the creation of man—to mark these differences and agreements with the present beings—and to point out, from the strata in which they exist, the order in which they probably were formed. The student, already delighted in the contemplation of surrounding creation, will be hereby led to another field of observation, where he will perceive traces of the vast changes which this planet has sustained, and will have his wonder and curiosity not a little excited by the remains of those beings which sojourned on this globe before man himself started into existence, at the call of the Almighty Architect. We recommend the book to all who feel an interest in the wonderful works of God, as manifested even in this speck of earth.*

---

23. Select Dissertations on various Subjects of Medical Science. By Sir GILBERT BLANE, Bart. Physician to the King. Octavo, pp. 380. T. and G. Underwood. 1820.

---

24. Elements of Pharmacy, and of the Chemical History of Materia Medica; containing an Explanation of the Chemical Processes of the London Pharmacopœia, on the different Theories received at present; the Chemical History of the several Articles of the Materia Medica of the London Pharmacopœia, and of some other Articles that have come into use since its publication; together with a Description of the most approved Furnaces actually used in the Practice of Chemistry, illustrated by Figures; and some Account of the Alchemical Speculations of the old Chemists. The whole attended as a Companion to the Author's General Treatise of Pharmacology. By S. F. GRAY, &c. &c.

### *Additional Subscribers since last Quarter.*

Belcher, Doctor—name but not residence left with Burgess & Hill
Burrows, Mr. Samuel, Surgeon, Bishopgate-street-within
Birch, William, Esq. (jun.)
Bowdoin College, United States
Baker, Mr. Park-lane, Camberwell
Brander, Mr. James M. Edinburgh
Brown, R. Esq. Fellow of the Royal College of Surgeons of London
Bell, Mr. Thomas, Assistant Surg. H.M.S. Impregnable, Plymouth
Callaway Mr. High-street, Southwark
Cavell, Mr. Henry, Assist. Surg. Dinapore
Corbet, David, M.D. Falkirk
Craw, Mr. Surgeon, Bombay Establishment
Cordukes, Mr. H. C. Burlington, Yorkshire
Cowen, H. Esq. M. D. Surgeon, 41st Regiment
Crisp, Mr. Surgeon, Northampton
Drummond, John, Esq. Surg. R. N. Grangemouth
Davies, Mr. William, Apothecary, Beckford-row, Walworth, Surry
Dix, Mr. Surgeon, Northampton
Dulhunty, John, Esq. late Surgeon of His Majesty's Naval Hospital at Plymouth, Russel-street, Bath
Fawcett, John, Esq. Surg. Sligo, Ireland
Forge, Francis, M. D. Great Suffield
Holme, Mr. William, Surg. &c. Barton, Lincolnshire
Hacket, William, M. D. Inspector of Health, Quebec
Howell, Mr. Wandsworth
Hamilton, C. M. Esq. Staples-Inn Buildings, Holborn
Hicks, Mr. Surgeon, Hodsden
Iliff, Mr. W. T. Surgeon, &c. Lambeth-road
Jagoe, Doctor, (name but not residence left with Burgess & Hill.)
Jones, Mr. John, Surgeon and Apothecary, Ilfracombe
Jones, J. J. Esq. Surg Hereford
Kerr, Dr. William, Northampton
Lucas, Mr. Surg. Church-street, Soho
Macintyre, W. (M.D.) Surg. R.N.
Moor, Mr. J. Surgeon, Castleton, Yorkshire
Mayer, Dr. Professor of Anatomy in the University of Bonn, on the Rhine; Corrrespondent for the North of Germany
Marsden, Mr. Wm. Chemist and Druggist, Holborn-hill
Marchant, Mr. Surgeon, North Curry, Somersetshire
Nicoll, Deputy Inspector of Hospitals, M.D.
Prall, (mis-stated Pratt) Mr. King-street
Porter, Mr. Thaddeus, Assist. Surg. Royal Navy, to Mediterranean
Pell, Mr. Surgeon, Alford
Richmond, Mr. John, Surgeon, &c. Grimsby, Linconshire
Roulston, Mr. Tooley-street
Root, Mr. G. Surgeon, Ross
Swan, Jos. Esq. Surgeon to the County Hospital, Lincoln
Schaw, James, Esq. Surgeon, R.N. Falkirk
Sinclair, Dr. Samuel, Royal Tyrone Regiment
Southwell, Mr. Oxford-street, Whitechapel
Stevens, Dr. Ely
Salmon, Wm. M.D. Read House, Gloucestershire
Waterworth, Mr. C. Surg. Dover-place, New Kent Road
Williams, Robert, Esq. Surg. Royal Navy, Tenby, South Wales
Wardrop, John, Esq. Surg. R. N. Falkirk
Walker, Dr. William, Richmond, Surrey
Wallas, Mr. Darlington
Welch, Mr. Apothecary to the Plymouth Dock Public Dispensary
Williams, D. (M D.) Liverpool
Williams, Mr. Surgeon, Wigton, Cumberland
Waller, Mr. Surg. Lewton, Bedfordshire

### REMOVALS.

Dr. James Veitch from Pimlico to Regent Street.

Dr. Salemi to Grenville Street, Brunswick Square.

